# Interactions of an *Arabidopsis* RanBPM homologue with LisH-CTLH domain proteins revealed high conservation of CTLH complexes in eukaryotes

**DOI:** 10.1186/1471-2229-12-83

**Published:** 2012-06-07

**Authors:** Eva Tomaštíková, Věra Cenklová, Lucie Kohoutová, Beáta Petrovská, Lenka Váchová, Petr Halada, Gabriela Kočárová, Pavla Binarová

**Affiliations:** 1 Centre of the Region Haná for Biotechnological and Agricultural Research, Institute of Experimental Botany AS CR, v.v.i., Sokolovská 6, Olomouc, 772 00, Czech Republic; 2 Institute of Experimental Botany, AS CR, v.v.i., Sokolovská 6, 772 00, Olomouc, Czech Republic; 3 Institute of Microbiology, AS CR, v.v.i., Vídeňská 1083, 142 20, Prague 4, Czech Republic

**Keywords:** Arabidopsis homologue of RanBPM, CTLH-complex, LisH-CTLH domain proteins

## Abstract

**Background:**

RanBPM (Ran-binding protein in the microtubule-organizing centre) was originally reported as a centrosome-associated protein in human cells. However, RanBPM protein containing highly conserved SPRY, LisH, CTLH and CRA domains is currently considered as a scaffolding protein with multiple cellular functions. A plant homologue of RanBPM has not yet been characterized.

**Results:**

Based on sequence similarity, we identified a homologue of the human RanBPM in *Arabidopsis thaliana.* AtRanBPM protein has highly conserved SPRY, LisH, CTLH and CRA domains. Cell fractionation showed that endogenous AtRanBPM or expressed GFP-AtRanBPM are mainly cytoplasmic proteins with only a minor portion detectable in microsomal fractions. AtRanBPM was identified predominantly in the form of soluble cytoplasmic complexes ~230 – 500 kDa in size. Immunopurification of AtRanBPM followed by mass spectrometric analysis identified proteins containing LisH and CRA domains; LisH, CRA, RING-U-box domains and a transducin/WD40 repeats in a complex with AtRanBPM. Homologues of identified proteins are known to be components of the C-terminal to the LisH motif (CTLH) complexes in humans and budding yeast. Microscopic analysis of GFP-AtRanBPM *in vivo* and immunofluorescence localization of endogenous AtRanBPM protein in cultured cells and seedlings of *Arabidopsis* showed mainly cytoplasmic and nuclear localization. Absence of colocalization with γ-tubulin was consistent with the biochemical data and suggests another than a centrosomal role of the AtRanBPM protein.

**Conclusion:**

We showed that as yet uncharacterized *Arabidopsis* RanBPM protein physically interacts with LisH-CTLH domain-containing proteins. The newly identified high molecular weight cytoplasmic protein complexes of AtRanBPM showed homology with CTLH types of complexes described in mammals and budding yeast. Although the exact functions of the CTLH complexes in scaffolding of protein degradation, in protein interactions and in signalling from the periphery to the cell centre are not yet fully understood, structural conservation of the complexes across eukaryotes suggests their important biological role.

## Background

Ras-related nuclear proteins (Rans) are abundant small GTPases, associated with Ran-specific nuclear GEFs (guanine nucleotide exchange factors), cytoplasmic GAPs (GTPase activating proteins), and with RanBP1 (Ran binding protein 1) that stimulate RanGTP hydrolysis in the cytoplasm [1,2]. The *Arabidopsis* genome contains three genes encoding AtRan [3], two genes encoding AtRanGAP related proteins [4] and three genes for RanBP1 isoforms – RanBP1a, RanBP1b and RanBP1c [3,5]. Plant Ran binding proteins (RanBPs) display significant homology with yeast and mammalian RanBPs, but there is little evidence for their biological function [6,7].

One RanBP in animal cells, RanBPM (RanBP9), was identified in a yeast two-hybrid screen with Ran as a bait. RanBPM comprises four domains – SPRY, LisH, CTLH and CRA and is homologous to the human RanBP10 protein [8]. Although RanBPM and RanBP10 have been shown to bind the Ran protein, they do not contain a consensus Ran-binding sequence [9]. RanBPM was defined as a member of the Scorpin family of proteins (SPRY-containing Ran binding protein) with a unique domain organization [10]. As reviewed in Suresh et al. [11], numerous protein interactions described for the RanBPM protein suggest its multiple roles in the regulation of protein stability, cell cycle regulation and other as yet undefined cellular processes.

RanBPM was reported to be a part of the large CTLH (C-terminal to the LisH motif) complexes [12-14]. CTLH complexes composed of LisH, CTLH and CRA domain containing proteins, transducin/WD40 repeat proteins, and armadillo repeat proteins have been found in mammals and yeast [15,16]. Mammalian and yeast CTLH complexes are structurally conserved but their biological function is still not fully understood. In yeast, the CTLH complex of Gid/Vid proteins plays a role in vacuole and proteasome-dependent fructose-1,6-bisphosphatase degradation [16]. Similarly, it has been suggested that CTLH complexes partake in lysosome and proteasome-dependent proteolysis in mammalian cells [17]. Data on proteins with SPRY, LisH, CTLH or CRA domain-containing proteins in plants are limited. In *Pinus radiata*, the SPRY domain containing protein, SEPR11, is the homologue of a Trithorax-group member and is involved in plant reproduction and development [18]. In *Oryza sativa,* the LisH domain-containing protein, OsLIS-L1 is required for male gametophyte formation and the first internode elongation [19].

Here we provide data on an *Arabidopsis* homologue of RanBPM that belongs to the uncharacterized family of plant SPRY, LisH, CTLH and CRA domain-containing proteins. We used *in silico* analysis, biochemical, proteomic and microscopic analyses *in vivo* and *in situ* to characterize AtRanBPM. We found that the AtRanBPM protein is present predominantly in the form of large cytoplasmic protein complexes that are structurally homologous to the CTLH type of complexes described in mammals and budding yeast.

## Results

### The Arabidopsis homologue of RanBPM is a SPRY-domain containing protein

By homology search of the *Arabidopsis thaliana* genome, we found a SPRY-domain containing protein AtRanBPM (At1g35470), which is a homologue of the human RanBPM (RanBP9) protein. The *AtRanBPM cDNA* contains a single open reading frame and consists of 467 amino acids. *AtRanBPM* is a member of the HOM002780 gene family that comprises 44 genes in 21 plant species, particularly from the ORTHO002658 subfamily. In *Arabidopsis* there are three paralogues of AtRanBPM (At4g09310, At4g09200, At4g09250**)** and one gene originating from the segmental duplication of chromosome 1 (At4g09340) (Plaza 2.0; A resource for plant comparative genomics, [20]). The products of these genes are described as SPRY domain-containing proteins but their biological roles in plants have not yet been identified.

**Figure 1:**
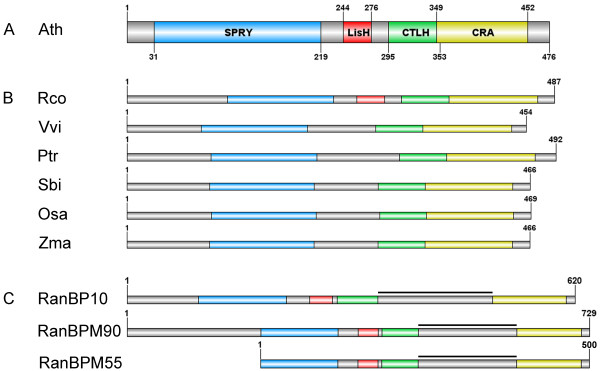
**Domain organization of*Arabidopsis*RanBPM protein and its plant and human homologues.A**- AtRanBPM protein consists of highly conserved SPRY, LisH, CTLH, and CRA domains. **B**- Comparison of AtRanBPM protein with its closest plant homologues. *Rco* – *Ricinus communis*, *Vvi* – *Vitis vinifera*, *Ptr* – *Populus trichocarpa*, *Sbi* – *Sorghum bicolor*, *Osa* – *Oryza sativa*, *Zma* – *Zea mays*. **C**- Comparison of AtRanBPM protein with its closest human homologues. Bold black lines indicate the 150 amino acids insertion between CTLH and CRA domains present only in human but not in plant RanBPM proteins. RanBPM90 – RanBPM of 90 kDa, RanBPM55 – RanBPM of 55 kDa.

The AtRanBPM protein is composed of four domains – SPRY, LisH, CTLH and CRA (Figure 1A). SPRY, LisH, CTLH and CRA domain-containing proteins are widely spread across eukaryotes. The protein encoded by *AtRanBPM* is annotated as a protein of unknown function (TAIR; The Arabidopsis Information Resource, [21]). As shown in Figure 1B, AtRanBPM has close homologues in other plant species such as *Ricinus communis* (67% identities, 79% positives), *Vitis vinifera* (66%, 78%), *Populus trichocarpa* (67%, 80%), *Sorghum bicolor* (57%, 74%), *Zea mays* (57%, 74%) and *Oryza sativa Japonica group* (57%, 73%) (Figure 1B, Additional file 1). The *Arabidopsis* RanBPM shows a close identity and similarity to its SPRY, LisH, CTLH and CRA domains with mammalian RanBPM and RanBP10 (Figure 1C, Additional file 2) (WU-BLAST, Basic Local Alignment Search Tool, [22]). However, there is an insertion of about 150 amino acids between the CTLH and CRA domains in human proteins (Figure 1C). A phylogenetic analysis performed using MEGA5 software (MEGA 5.02, Molecular Evolutionary Genetics Analysis, [23]) revealed that *Arabidopsis* RanBPM grouped with homologues from other plant species. The metazoan RanBPM homologues grouped in a separated clade. The *Saccharomyces* homologue, Gid1/Vid30, appears to be ancestral to the plant and metazoan clades (Additional file 3).

SPRY, LisH, CTLH and CRA conserved domains are mainly involved in protein-protein interactions. The SPRY domain (SPla and the RYanodine Receptor) was originally identified as a structural motif in ryanodine receptors [24]. Proteins with SPRY domains are involved in intracellular calcium release, as is the case for ryanodine receptors, in RNA metabolism and in regulatory and developmental processes [25]. The LisH domain (Lissencephaly type-1-like homology motif) functions as a stable homodimerization and dimerization motif and its contribution to the dynamics of microtubules has been suggested [26]. Located adjacent to the C-terminus of the LisH domain is a C-terminal to the LisH motif (CTLH) that has a putative α-helical structure with an as yet undescribed function. The CRA domain (CT11-RanBPM) was found to be a motif in the C-terminal part of RanBPM and RanBP10 and represents a protein-protein interaction domain often found in Ran-binding proteins [27].

**Figure 2:**
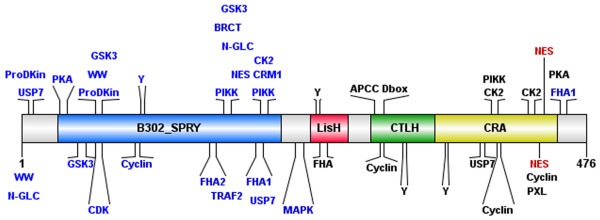
**Putative interaction sites and binding motifs in AtRanBPM amino acid sequence.** To predict putative interaction sites and binding motifs ELM [28] and SUMOsp 2.0 [29] databases were used.

Putative interaction sites of AtRanBPM were analysed using ELM (The Eukaryotic Linear Motif for functional sites in proteins, [28]) and SUMOsp 2.0 databases (SUMOylation Sites Prediction, [29]) (Figure 2). There are common motifs in the AtRanBPM amino acid sequence, such as phosphorylation consensus sites for phosphoinositide-3-OH kinase related kinases, a protein kinase A phosphorylation site, a mitogen-activated protein kinase phosphorylation site and docking motif, and a putative cyclin recognition site. Further, the AtRanBPM amino acid sequence possesses forkhead-associated phosphopeptide binding domains and a Y-based sorting signal which might act in endosomal and secretory pathways.

### Protein expression levels, cellular distribution and molecular forms of AtRanBPM

The AtRanBPM coding sequence [EMBL:AEE31799] was cloned into Gateway-compatible plant binary vectors for N-terminal GFP and C-terminal GFP fusion. *Arabidopsis* cell suspension cultures were transformed according to Mathur et al. [30]. Transformed *Arabidopsis* plants were obtained through the floral-dip method [31].

**Figure 3:**
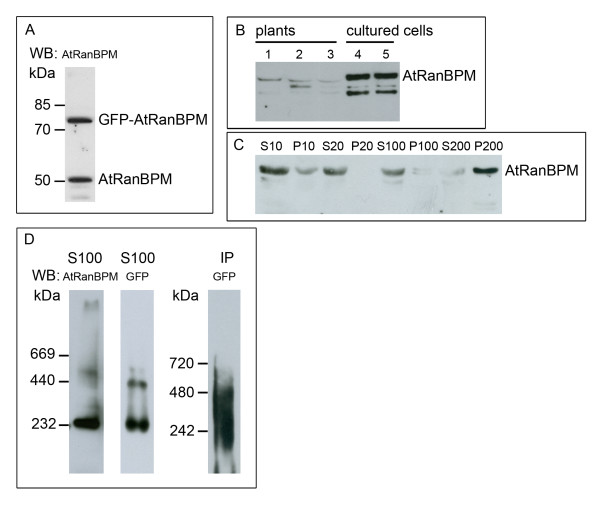
**Cellular distribution of AtRanBPM and AtRanBPM complexes.A**- The antibody against AtRanBPM recognizes a band of 52 kDa, which corresponds to endogenous AtRanBPM protein, and a band of 80 kDa, which corresponds to expressed GFP-AtRanBPM from cultured cells expressing GFP-AtRanBPM. **B**- Comparison of protein levels of AtRanBPM in *Arabidopsis* seedlings and cultured cells. Lines 1, 2, and 3 – fractions S10 from four-, eight-, and 15-d-old seedlings; lines 4 and 5 – fractions S10 from exponentially growing cultured cells (3 days after subculture) and stationary phase cultured cells (6 days after subculture). Per line, 25 μg of protein content of S10 fraction was loaded. **C**- Distribution of AtRanBPM in the cellular fractions of *Arabidopsis* cultured cells. The majority of the protein was present in cytoplasm (fractions S10, S20, S100), a small amount of the protein was found in pellet P10. AtRanBPM was spun down to high-speed pellet P200 probably in the form of complexes. **D**- GFP-AtRanBPM complexes from high-speed supernatant S100 and from immunoprecipitate (IP) were resolved by non-denaturing PAGE and detected by anti-AtRanBPM antibody or anti-GFP antibody as indicated. For S100 fractions, 15 and 10 μg of protein content was loaded.

An antibody against a peptide from the C-terminal part of the AtRanBPM molecule was produced in rabbits and affinity purified using the immunogenic peptide. When cell lines expressing GFP-AtRanBPM were analysed by Western blotting of separated *Arabidopsis* extracts, the antibody recognized a band of 52 kDa, the predicted molecular mass (MW) of AtRanBPM, and a band corresponding to the MW of GFP-AtRanBPM (Figure 3A). Western blot further showed that the protein levels of AtRanBPM were much lower in *Arabidopsis* seedlings than cell cultures. In cultured *Arabidopsis* cells, there was no significant difference in AtRanBPM protein levels between dividing cells and stationary growing non-dividing cells (Figure 3B). Similarly, there was no difference in AtRanBPM protein levels between four, eight and 15 day old *Arabidopsis* seedlings. Our data correspond to those on AtRanBPM expression found in the publically available Affymetrix expression databases, the Arabidopsis eFP browser [32], and the Genevestigator database [33] where AtRanBPM has a constant level of expression in analysed tissues as well as during seedling development.

The cellular distribution of AtRanBPM was analysed using differential centrifugation. As shown in Figure 3C, the majority of the protein was cytoplasmic and only small amounts sedimented either in low speed pellets or in the microsomal pellet (P100). The fact that AtRanBPM was also sedimented from a high-speed cytoplasmic S100 extract to pellet P200 indicated that the protein might be present in the form of higher MW complexes. The separation of S100 high speed extract by native polyacrylamide gel electrophoresis (native PAGE) confirmed cell fractionation data and suggested that AtRanBPM is a component of protein complexes with a molecular mass of around ~230 – 500 kDa (Figure 3D). Further, we immunopurified GFP-AtRanBPM using the GFP trap. Separation of purified GFP-AtRanBPM by native PAGE followed by Western blotting showed the presence of complexes of similar size as observed when high-speed supernatant S100 was analysed (Figure 3D). Altogether these data showed that AtRanBPM is present predominantly in the form of large cytoplasmic complexes.

### Analysis of AtRanBPM protein complexes by mass spectrometry

**Figure 4:**
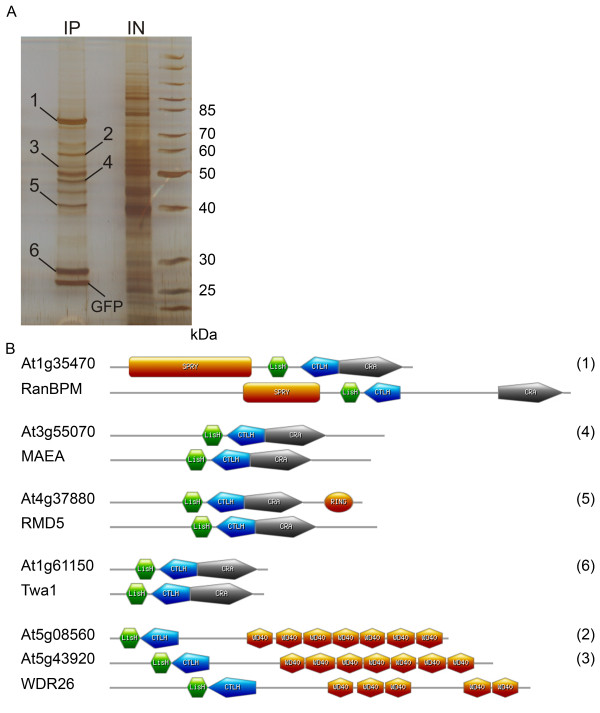
**Proteins of CTLH complexes copurified with AtRanBPM from*Arabidopsis*extracts.A**- Affinity purification of GFP-AtRanBPM from the input of S20 of GFP-AtRanBPM culture (IN) using GFP-Trap A beads. Proteins were eluted from affinity beads (IP: anti-GFP) and run on SDS gel electrophoresis with silver staining detection. MALDI-MS analysis identified among proteins copurified with AtRanBPM the plant homologues of proteins that form CTLH complex in human cells. **B**- Comparison of proteins copurified with AtRanBPM (numbers in brackets present number of band shown in Figure 4A) with their counterparts - the proteins of human CTLH complex.

To analyse putative interactors of AtRanBPM in the complexes, GFP-AtRanBPM and copurified proteins were separated by SDS-PAGE followed by silver staining, and specific bands were excised and analysed by MALDI-MS (Figure 4A). GFP immunopurification performed in extracts from wild type cell was used as a negative control. Neither AtRanBPM protein nor bands corresponding to the MW of proteins copurified with GFP-AtRanBPM were observed in the negative controls and only background contamination such as HSPs and cytoskeletal proteins were detected by MALDI-MS (Additional file 4). We found that proteins reproducibly copurifying with AtRanBPM belong to CTLH-domain containing proteins including LisH (At1g61150), CRA and U-box (At3g55070, At4g37880), and to the transducin/WD-40 domain-containing proteins At5g08560, and At5g43920 (Table 1, Figure 4B). Alternatively to MALDI-MS, we used LC-MALDI-MS/MS analysis of eluates from the GFP trap to confirm the interaction of GFP-AtRanBPM with the proteins mentioned above.

**Table 1:** Proteins copurified with AtRanBPM are members of CTLH complexes

Protein name	AGI number	Band No.	MW [kDa]	No. peptides	Sequence coverage [%]	Aranet/ATTED-II	Members of CTLH complex in other organisms
AtRanBPM	At1g35470	1	52	17/41	41/60		RanBPM [Q96S59] Gid1/Vid30 [P53076]***
LisH and RanBPM domain-containing protein	At1g61150	6	25	8/26	41/82	-/+	Twa1 [Q9NWU2]**Gid8 [P40208]***
LisH/CRA/RING-U-box domain-containing protein	At3g55070	4	48	10/17	28/31	+/-	MAEA [Q7L5Y9]*Gid9p [P40492]***
LisH/CRA/RING-U-box domain-containing protein	At4g37880	5	44	9/8	32/22	+/-	RMD5 [Q9H871]*Gid2 [Q12508]***
Transducin/WD40 domain-containing protein	At5g08560	2	66	17/14	38/31	+/-	WDR26 [Q9H7D7]
Transducin/WD40 domain-containing protein	At5g43920	3	60	6/4	13/7	+/-	WDR26 [Q9H7D7]

Proteins (for band numbering see Figure 4A) copurified with AtRanBPM protein were identified by both MALDI-MS and LC-MALDI-MS/MS analysis. The values before and after the slash in the columns “No. peptides” and “Sequence coverage” refer to MALDI-MS and LC-MALDI-MS/MS data, respectively. Homologues of human CTLH-complex members reported by Kobayashi et al., [15] (*), proteins in the complex described by Umeda et al. [12] (**), yeast homologues in the Gid complex published by Regelmann et al. [16] (***).

Database search indicated that the proteins copurified with AtRanBPM were homologous to protein components of the human CTLH complex [15]. The CTLH complex is annotated in CORUM (The comprehensive resource for mammalian complexes, [34]) as a complex with a putative function in regulating cell migration, nucleokinesis, chromosome segregation and microtubule dynamics. Components of the CTLH complex are conserved amongst animal species and their presence is predicted for other eukaryotes such as plants and fungi. A relatively lower degree of homology with individual mammalian and plant members of CTLH complexes (Figure 4B, Additional file 5) is due to the fact that part of the molecule separating highly conserved domains contains insertions and deletions of various lengths, differing among plant, mammalian and/or yeast homologues.

The LC-MALDI-MS/MS analysis also identified among proteins copurified with AtRanBPM Armadillo-repeat-containing protein (At3g08947) (Additional file 6). However, the protein was not proven to be a homologue of the human armadillo repeat ARMC8, a member of the CTLH complex [34,35]. Further, Yippee family proteins (At5g53940, At2g40110) and Yippee-like protein (At3g08890) were copurified with AtRanBPM and identified by LC-MALDI-MS/MS (Additional file 6). Yippee-like proteins belong to the YPEL family of proteins whose members have been described as interactors of the human RanBPM [10]. Proteins At5g08560, At5g43920, At3g55070, and At3g37880, copurified with AtRanBPM were predicted to interact within a functional network in the Aranet database (Probabilistic Functional Gene Network for Arabidopsis thaliana, [36]) (Table 1).

Together these data suggest that plant AtRanBPM protein, like its yeast and mammalian homologues, is a component of a cytoplasmic multiprotein complex where it interacts with LisH-CTLH domain-containing proteins and armadillo-repeat-containing proteins.

### In vivo localization of GFP-AtRanBPM and AtRanBPM immunofluorescence labelling in Arabidopsis cultured cells and seedlings

**Figure 5:**
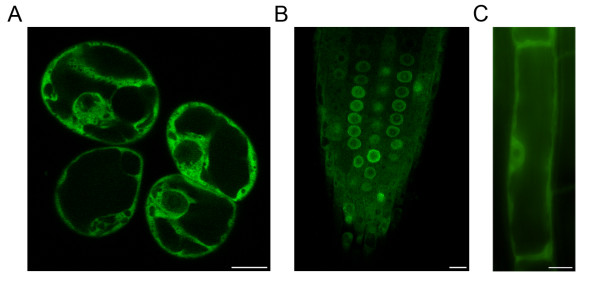
***In vivo*localization of GFP-AtRanBPM in*Arabidopsis*cells and seedlings.A**- Localization of GFP-AtRanBPM in *Arabidopsis* suspension culture. The protein is localized in the cytoplasm and in the perinuclear area. Weaker signal is present in nuclei. **B**- Root tip of 8 days old *Arabidopsis* plant. Weak cytoplasmic signal for GFP-AtRanBPM is enriched in the perinuclear area. **C**- Cytoplasmic and perinuclear localization of GFP-AtRanBPM in differentiated cells of root. Bars = 10 μm.

In cultured cells of *Arabidopsis,* the GFP-AtRanBPM signal was localized in the cytoplasm and in the perinuclear area of interphase cells, with a weaker signal detected in nuclei (Figure 5A). We observed no gain-of-function phenotype of *Arabidopsis* seedlings expressing GFP-AtRanBPM from Gateway vectors. Similarly to cultured cells, the GFP-AtRanBPM signal was localized in the cytoplasm, in nuclei and accumulated in the vicinity of nuclei in dividing zone of roots (Figure 5B). In differentiated root cells, GFP-AtRanBPM was cytoplasmic and in the perinuclear area (Figure 5C, Additional file 7). C-terminal GFP fusions showed a similar localization pattern when expressed transiently in cultured cells of *Arabidopsis* (Additional file 8).

**Figure 6:**
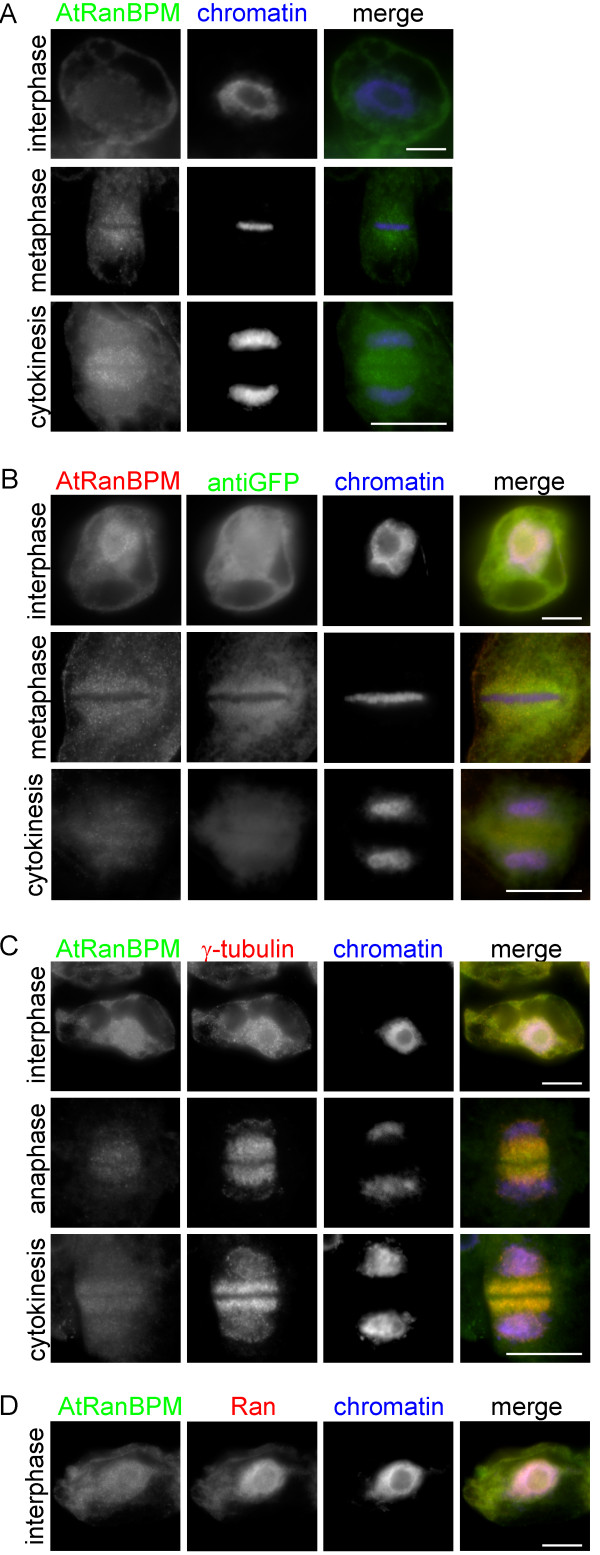
**Immunolocalization of AtRanBPM in*Arabidopsis*cultured cells.A**- Immunolocalization of endogenous AtRanBPM with affinity purified anti-AtRanBPM antibody. Signal for AtRanBPM was cytoplasmic with slight accumulation in the vicinity of nuclei and in dividing cells with slight enrichment in spindle and phragmoplast area. (AtRanBPM – green, chromatin – blue). **B**- Double immunofluorescence analysis of cells expressing GFP-AtRanBPM confirmed similar localization pattern for expressed GFP protein and for endogenous AtRanBPM protein (Figure 6A) (GFP-AtRanBPM – green, endogenous AtRanBPM – red, chromatin – blue). **C**- Double immunofluorescence analysis of AtRanBPM (green) and γ-tubulin (red) did not confirm colocalization of both proteins in interphase and in dividing cells. **D**- Double immunofluorescence of AtRanBPM (green) and Ran (red). Only a small portion of AtRanBPM is localized in nuclei compared to intensive nuclear signal for Ran protein. Bars = 10 μm.

Immunofluorescence analyses showed that the AtRanBPM protein was distributed patchily in the cytoplasm and nuclei; in non-dividing cells it accumulated in the perinuclear region and in dividing cells, the signal was slightly enriched in the area of the mitotic spindle and the phragmoplast (Figure 6A). Further we compared localization of the endogenous protein and GFP-AtRanBPM by double labelling with anti-AtRanBPM and anti-GFP antibodies. We confirmed a similar localization pattern for AtRanBPM and expressed AtRanBPM with a cytoplasmic signal slightly enriched in the perinuclear area and a weaker nuclear signal (Figure 6B).

To determine whether the AtRanBPM protein plays a role in microtubule organization like the truncated version of human RanBPM [37], we studied the colocalization of AtRanBPM with the centrosomal protein, γ-tubulin. In acentrosomal plant cells, γ-tubulin is present with dispersed sites of microtubule nucleation in the cytoplasm, with nuclei, and on membranes and microtubules [38]. We found that the *Arabidopsis* homologue AtRanBPM did not colocalize with γ-tubulin-positive sites either present with the nuclear envelope or on microtubular arrays (Figure 6C).

As a portion of RanBPM was reported to be associated with the Ran protein in mammalian cells [14] we performed double immunofluorescence labelling of AtRanBPM and Ran protein. In cultured cells of *Arabidopsis,* Ran was localized in the cytoplasm and in nuclei but only a small fraction of AtRanBPM colocalized with nuclear Ran.

## Discussion

RanBPM protein was first characterized in human cells as a centrosomal protein involved in microtubule nucleation which colocalized with γ-tubulin at centrosomes and at ectopic nucleation sites [37]. The data on RanBPM initiated an investigation of the role of the RanGTPase pathway and its role in chromatin-mediated microtubule nucleation and spindle assembly [39]. However, later it was found that antibody against the 55 kDa form of RanBPM that was characterized by Nakamura et al. [37] did not recognize the full length 90 kDa RanBPM protein. The truncated form of RanBPM (55 kDa) was shown to be an incorrectly translated product of the *RanBPM* gene and moreover, only the truncated version but not the whole RanBPM molecule was active in microtubule nucleation [14]. The complete RanBPM molecule thus does not show the same properties as the truncated version and it was depicted as a scaffold protein that links and modulates interactions between cell surface receptors and their intracellular signalling pathways [11,40]. The molecular mass of plant AtRanBPM (52 kDa) corresponds to that of the truncated RanBPM rather than to the whole human RanBPM molecule. However, the reason for the apparent discrepancy in size of the full length human and plant RanBPM molecules is an insertion of 150 amino acids between CTLH and CRA domains in the plant protein and the presence of a long stretch of proline and glutamine residues on the N-terminal part of human RanBPM molecule.

We found that AtRanBPM maintained the properties of the full length RanBPM of mammals: (i) while the truncated version of human RanBPM is present in centrosomal and ectopic microtubule nucleation sites [37], AtRanBPM protein in acentrosomal plant cells did not colocalize with microtubule nucleation sites positive for γ-tubulin; (ii) proteins of the γ-tubulin nucleating machinery were not identified by MALDI-MS or LC-MALDI-MS/MS among proteins interacting with AtRanBPM, nor were they found in coexpression databases and a biological role of AtRanBPM other than in processes relating to centrosomes and microtubules was thus suggested; (iii) the presence of highly conserved SPRY, LisH, CTLH and CRA domains in AtRanBPM indicated its function in mediating multiple protein interactions that were described for the whole molecule of human RanBPM [12,14,15].

AtRanBPM protein was not specifically enriched with microtubules in dividing cells or localized in putative microtubule nucleation sites. Subcellular localization of AtRanBPM corresponded to published data on the subcellular localization of the whole RanBPM molecule in mammalian cells [14,41,42]. Since a weak nuclear signal, observed for human RanBPM was suggested to be a consequence of over-expression of its tagged version [40], we analysed by immunofluorescence the nuclear localization for endogenous AtRanBPM protein. A smaller portion of AtRanBPM was present in nuclei and the possibility that the nuclear signal observed for GFP-AtRanBPM or AtRanBPM-GFP resulted from over expression of GFP fusions was thus excluded. We observed only partial colocalization of Ran and AtRanBPM in nuclei. It would be interesting to address the question whether transport of the AtRanBPM complex between nucleus, perinuclear area and cytoplasm might be regulated by a weak interaction between AtRanBPM and Ran as it was suggested for mammalian RanBPM [14]. Heterogeneity in the localization of mammalian RanBPM might depend on interacting proteins [15]. Conserved domains of RanBPM protein are responsible for interactions and complex formations with a physiologically divergent group of proteins. As reviewed by Suresh et al. [11], RanBPM is a modulator/protein stabilizer, a regulator of transcriptional activity, and has cell cycle and neurological functions. RanBPM interacts with a wide range of receptors [43], acts as a scaffolding protein [44,45], and is involved in signal transduction pathways [44,46], and the apoptotic pathways [47]. Plant homologues of *RanBPM* belong to genes with unknown functions. We found that the product of *AtRanBPM* is predominantly a cytoplasmic protein that is a part of protein complexes with a molecular mass of approximately 230 – 500 kDa. A large protein complex of RanBPM was described by Nishitani et al. [14] and Ideguchi et al. [13]. Later the complex was designated as a CTLH complex [15]. Five from eleven CTLH domain-containing proteins that were identified in databases were described as being a part of the CTLH complex and an interaction within the complex via LisH-CTLH domains was suggested [15]. In *Saccharomyces cerevisiae* all four CTLH domain-containing proteins present in the genome are part of the CTLH-like complex Gid. The Gid complex is suggested to be involved in proteasome-dependent glucose-induced degradation of fructose-1,6-bisphosphatase [16,48]. There are twenty one CTLH-domain containing proteins encoded in the *Arabidopsis* genome and of these, six proteins were identified in our experiments to form CTLH-like complexes with AtRanBPM protein. Though we identified the same spectrum of AtRanBPM interacting proteins in several independent experiments it cannot be ruled out that the members of CTLH complexes exist and remain to be identified. We found that the antibody raised against a peptide from the AtRanBPM sequence did not work in immunopurification experiments. The immunopurifications performed with a panel of antibodies against the AtRanBPM protein might help to disclose the presence of other putative members of plant CTLH complexes. Alternatively, immunopurification of the CTLH complex or pull down experiments via proteins that copurified with AtRanBPM might extend our knowledge of the composition and protein interactions of newly identified CTLH complexes.

All the members of the CTLH complexes that we identified belong to yet uncharacterized plant LisH-CTLH domain-containing proteins. Protein At1g61150 is a homologue of the human Twa1 protein found in yeast by two-hybrid analysis to interact with RanBPM [12]. LisH, CRA, and RING/U-box domain-containing proteins of unknown function (At3g55070, At4g37880), copurified with AtRanBPM, are homologous to human MAEA and RMD5, respectively, that are members of the CTLH complex annotated by Kobayashi et al. [15]. Similarly, LisH and CTLH domain-containing proteins such as RanBP10 or the WD repeat domain 26 (WDR26), were suggested to be putative members of the CTLH complex [15]. We found that uncharacterized *Arabidopsis* transducin/WD-40 domain-containing proteins At5g08560 and At5g43920 which copurified with AtRanBPM, are homologues of human WDR26 protein and thus belong to components of the plant CTLH complex. Corresponding to our microscopic data on cytoplasmic and nuclear localizations of AtRanBPM, the members of the human CTLH complex, proteins RanBPM, RMD5, WDR26, Twa1, and MAEA, were shown to have predominantly cytoplasmic and a weaker nuclear localization [12,15,49].

A component of the human CTLH complex, the armadillo repeat-containing protein ARMC8, when exogenously expressed, upregulates the proteolytic degradation of ectopically expressed α-catenin, and thus was suggested to play an important role in the ubiquitin-independent and proteasome-dependent degradation of α-catenin [35]. α-Catenin is known to function as a link protein between cadherins and actin-containing filaments of the cytoskeleton [50]; thus a complex might regulate molecular adhesion. We identified Armadillo-repeat-containing protein (At3g08947) among proteins copurified with AtRanBPM but its homology with armadillo repeat ARMC8, a member of the human CTLH complex, was not proven. However, we found that expression of copurified protein At4g37880, a homologue of RMD5 protein, correlated strongly with expression of the armadillo repeat-containing protein At3g01400, one of the homologues of ARMC8, and with expression of the plant adhesion molecule RabGAP/TBC domain-containing protein At3g02460 (ATTED-II, [51]). It is tempting to speculate whether similar function in connecting external signals in adhesion with the cell centre analogous to the cadherin and catenin pathway in animals might exist for plant CTLH complexes. However, further experimental data are needed to prove this hypothetical function of the CTLH complexes in plants.

Besides copurification with CTLH complex proteins, we found that plant AtRanBPM interacts with Yippee proteins (At5g53940, At2g40110) and Yippee-like protein (At3g08890). It was shown in human cells that RanBPM binds members of YPEL (Yippee like) family of proteins involved in a cell division-related functions [10]. Our data showed that binding with Yippee proteins might present one of the AtRanBPM multiple protein interactions in plant cells too.

## Conclusions

RanBPM protein was shown to have numerous apparently functionally unrelated protein interactors, including membrane receptors, cytoplasmic and nuclear proteins [12,15]. This suggests its role in a variety of cellular processes. Our biochemical and proteomic studies showed that AtRanBPM is a component of plant homologues of CTLH complexes. The fact that CTLH complexes are present in mammals, yeast and in plants suggests their structural conservation in evolution. CTLH complexes thus might represent a module with important but not fully understood biological functions.

## Methods

### Plant material

*Arabidopsis thaliana* (*L*.) *Heynh*. plants, ecotype Columbia were used. Sterilized seeds were sown and stratified at 4 °C for 2 days, then grown under 8 h of light, 16 h of darkness at 20 °C and after 4 weeks, under 16 h of light and 8 h of darkness. Seeds were cultivated on half-strength Murashige and Skoog (MS) medium (Duchefa), supplemented with 0.25 mM MES, 1% saccharose and 1% phytoagar. Transformed seedlings were selected as described in Harrison et al. [52]. *Arabidopsis thaliana* suspension cultures were cultured as described in Drykova et al. [38].

### Molecular cloning of AtRanBPM

AtRanBPM coding sequence [EMBL:AEE31799] was obtained by PCR amplification using *Arabidopsis thaliana* cDNA as template and Platinum Pfx DNA Polymerase (Invitrogen). The PCR primers with *attB* sites (underlined) were designed according to the manufacturer’s intruction, forward 5’ – GGGGACAAGTTTGTACAAAAAAGCAGGCTTCATGAACTCTTCACCACCACCG – 3’, reverse 5' – GGGGACCACTTTGTACAAGAAAGCTGGGTCTTAGTCTCCATTCAGTGACCGCCTTTC – 3’ for N-terminal GFP fusion and for C-terminal GFP fusion forward 5' – GGGGACAAGTTTGTACAAAAAAGCAGGCTTCATGAACTCTTCACCACCACCG – 3’, reverse 5' – GGGGACCACTTTGTACAAGAAAGCTGGGTCTTAGTCTCCATTCAGTGACCGCCTTTC – 3´. PCR products were isolated using QIAquick gel extraction kit (Qiagen) and afterwards cloned by Gateway technology (Invitrogen). Gateway binary vectors pK7WGF2,0 for N-terminal GFP fusion, pMDC43 for C-terminal GFP fusion [53] were used.

### Stable transformation of cell suspension cultures and plants

*Arabidopsis* suspension cultures of Landsberg erecta ecotype stably expressing GFP-AtRanBPM or transiently expressing AtRanBPM-GFP were prepared according to the protocol of Mathur et al. [30]. Arabidopsis plants Landsberg erecta ecotype were transformed by the floral-dip method [31].

### Preparation of protein extracts and its fractionation by differential centrifugation

Cultured suspension cells or seedlings of *Arabidopsis thaliana* were ground in liquid nitrogen and thawed in extraction buffer in ratio 1:1 weight to volume. Extraction buffer was 50 mM Na-Hepes, pH 7.5, 75 mM NaCl, 1 mM MgCl_2_, 1 mM EGTA, 1 mM DTT supplemented with inhibitors of proteases and phosphatases according to Drykova et al. [38] with increased concentration of 4-(2-aminoethyl)benzenesulfonyl fluoride hydrochloride to 2 mM and β-glycerophosphate to 60 mM. Crude extracts were centrifuged at 10,000 *g* for 10 min at 4 °C; obtained supernatants S10 were subsequently centrifuged at 20,000 *g* for 0.5 h at 4 °C to gain supernatants S20 and pellets P20. Supernatants S20 were then centrifuged at 100,000 *g* for 1 h at 4 °C when S100 were needed. Supernatants S100 were centrifuged at 200,000 *g* for 1.5 h at 4 °C to gain supernatants S200 and pellets P200 when needed. For differential centrifugation, pellets were resuspended in a volume of extraction buffer equal to the volume of the corresponding supernatants; equal sample volumes were loaded on SDS-PAGE gel.

### Immunoprecipitation of GFP-AtRanBPM

Cell extracts S20 (~ 4 mg protein/mL) were used directly or solubilized by 1% NP-40 (Calbiochem) for 1 h at 4 °C. Immunoprecipitations of GFP-AtRanBPM protein were then performed using GFP-Trap_A (ChromoTek) according to the manufactures’ instructions. GFP immunopurification from extracts S20 of wild type Ler *Arabidopsis* culture was used as a negative control.

### Protein digestion and mass spectrometry

The analysed protein bands were cut out of the Ag-stained SDS-PAGE gels, trypsin-digested and the proteins were identified by peptide mass mapping as described elsewhere [54]. Alternatively, eluates from the GFP trap were denatured in 8 M urea and digested overnight using trypsin. After desalting the resulting peptides were separated using the Ultimate HPLC system (LC Packings) on a Magic C18AQ column. The eluent from the column was spotted directly onto PAC-target using a ProteineerFC spotting device (Bruker Daltonics). Automatic MALDI MS/MS analyses were performed on an Ultraflex III TOF/TOF (Bruker Daltonics) and the proteins were identified by searching MS/MS spectra against *A. thaliana* subset of NCBI database using the in-house Mascot program (Matrixscience). The high resolution MALDI spectra were acquired on an APEX-Qe 9.4 T FTMS instrument (Bruker Daltonics).

### Electrophoresis and immunoblotting

Protein samples after addition of appropriate sample buffer were separated either on 10% SDS-PAGE gels [55] or on 3-10% non-denaturing PAGE gels as described previously in Drykova et al. [38]. Proteins were transferred onto 0.45 μm polyvinylidene fluoride membranes (Millipore) by wet electroblotting and immunodetected with appropriate antibody. Anti-AtRanBPM antibody was designed against peptide of AtRanBPM amino acid sequence (CSTNPNKKDVQRS), raised in rabbits and purified with immunogenic peptide coupled to protein A sepharose beads (GeneScript) and used in dilution 1:100 for Western blotting from non-denaturating PAGE, 1:300-1:500 for Western blotting from SDS-PAGE. Anti-GFP ab290 (Abcam) was diluted 1:2,000. Secondary anti-Rabbit IgG ECL Antibody, HRP Conjugated (GE Healthcare) was diluted 1:10,000; Super Signal West Pico Chemiluminiscent Substrate (Thermal Scientific) was used according to manufacturer’s instructions. Alternatively, SDS-PAGE gels were stained with silver, and the selected protein bands analysed by MALDI-MS.

### Immunofluorescence

*Arabidopsis thaliana* suspension cultures were fixed for 30 min using 3.7% paraformaldehyde and processed for immunofluorescence labelling according to Binarová et al. [56]. Primary antibodies used: mouse anti-α-tubulin monoclonal antibody DM1A (Sigma) diluted 1:500, mouse monoclonal anti-γ-tubulin TU-32 (kindly provided by Pavel Dráber, IMG, Prague, Czech Republic) diluted 1:10, mouse anti-GFP antibody (Abcam) diluted 1:1,000, and mouse antibody against human Ran protein (BD Transduction Laboratories) diluted 1:1,000. Anti-AtRanBPM antibody was used in a dilution 1:1,000, anti-rabbit or anti-mouse secondary antibodies DyLight 488 or DyLight 549 (Jackson Immuno Research Laboratories) were diluted 1:250. DNA was stained with DAPI.

### Microscopy

Microscopical analysis was performed on an IX81 motorized inverted research microscope CellR (Olympus) equipped with DSU (Disk Scanning Unit) and digital monochrome CCD camera CCD-ORCA/ER. To avoid filter crosstalk, fluorescence was detected using HQ 480/40 exciter and HQ 510/560 emitter filter cubes for DyLight 488 and HQ 545/30 exciter and HQ 610/75 emitter filter cubes for DyLight 549 (both AHF AnalysenTechnique). Confocal images were taken on Olympus FluoView FV1000 based on IX81 microscope using PLAPO 100x/1.45 and UPLSAPO 60x/1.35 objectives. GFP was excited by 473 nm solid state laser and its emission was detected from 485 to 545 nm. Images were processed and analysed using CellR and FV10-ASW (Olympus). Adobe Photoshop 7.0 was used for preparation of figures.

### Database search

To identify putative domains, SMART (Simple Modular Architecture Research Tool, [57], http://smart.embl-heidelberg.de/) and Pfam ([58], http://pfam.sanger.ac.uk/) databases were used. Multiple sequence alignment was performed by ClustalX2 [59] and evaluation of similarity and identity was done by WU-BLAST (Basic Local Alignment Search Tool, [22], http://www.ebi.ac.uk/Tools/sss/wublast/). Schematic drawings of protein structure were prepared in DOG (Domain Graph, [60]) and MyDomains (Prosite, http://prosite.expasy.org/cgi-bin/prosite/mydomains/). Search for putative interaction sites in AtRanBPM amino acid sequence was done in ELM (The eukaryotic linear motif resource for functional sites in proteins, [28], http://elm.eu.org/) and SumoSP 2.0 (Sumoylation sites prediction [29], http://sumosp.biocuckoo.org/). Phylogenetic relationships were analysed in MEGA 5.02 (Molecular evolutionary genetic analysis, [23]). Genes with similar expression pattern to AtRanBPM were identified in publically available databases ATTED-II ([51], http://atted.jp/) and AraNet ([36], http://www.functionalnet.org/aranet/). Putative interactors were analysed by Arabidopsis eFP browser ([32], http://bar.utoronto.ca/efp/cgi-bin/efpWeb.cgi) and Genevestigator ([33], https://www.genevestigator.com/gv/plant.jsp). The information and phylogenetic relationships of human CTLH complex with similar complexes from other species were found in CORUM ([34], http://mips.helmholtz-muenchen.de/genre/proj/corum/).

## Abbreviations

CRA, CT11-RanBPM; CTLH, C-terminal to LisH; GAP, GTPase activating protein; GEF, Guanine nucleotide exchange factor; GFP, Green fluorescent protein; Gid/Vid proteins, Glucose-induced degradation/vacuole-induced degradation proteins; LC, Liquid chromatography; LisH, Lissencephaly type-1-like homology; RanBP, Ran binding protein; RanBP1, Ran binding protein1; RanBPM, Ran binding protein in microtubule organizing centre; SPRY, SPIa and Ryanodine receptor; Scorpin family, SPRY-containing Ran binding protein family.

## Competing interests

The authors declare that they have no competing interests.

## Authors' contributions

ET performed databases search, *in vivo* microscopical analyses and wrote the manuscript. VC carried out immunolocalization and microscopy, and participated in writing the manuscript. LK performed biochemical analysis, isolation of protein complexes and confocal microscopy, BP performed cloning and together with LV, created stably transformed cell lines and plants of *Arabidopsis thaliana*, and performed *in vivo* microscopical analyses. PH performed proteomic analyses. GK undertook immunopurification of protein complexes. PB designed and coordinated experimental plans, and wrote the manuscript. All authors read and approved the final manuscript.

## Supplementary Material

Additional file 1 **Multiple sequence alignment of AtRanBPM plant homologues.** Sequence alignment of *Arabidopsis* RanBPM with *Ricinus communisVitis viniferaPopulus trichocarpaSorghum bicolorOryza sativa* and *Zea mays* homologues. Alignment was done using ClustalX2 software [59]. Sequence data of this alignment can be found at accession numbers [Swiss-Prot:F4HYD7] for At1g35470, [Swiss-Prot:B9S762] for *R. communis*, [Swiss-Prot:F6HWC3] for *V. vinifera*, [Swiss-Prot:B9MWC1] for *P. trichocarpa*, [Swiss-Prot:C5XUT1] for *S. bicolor*, [Swiss-Prot:Q6ZI83] for *O. sativa*, [Swiss-Prot:B6UAR9] for *Z. mays*. Click here for file

Additional file 2 **Identities and similarities of conserved domains between Arabidopsis RanBPM and its human homologues.** Sequence alignment was done for conserved domains SPRY (A), LisH (B), CTLH (C) and CRA (D) of full-sized 90 kDa form (RanBPM_90) and 55 kDa form (RanBPM_55) of human RanBPM protein, and RanBP10 with *Arabidopsis* RanBPM using ClustalX2 software [59]. Sequence data of this alignment can be found at accession numbers [Swiss-Prot:Q6VN20] for RanBP10, [Swiss-Prot:Q96S59] for 90 kDa RanBPM and [EMBL:BAA23216] for 55 kDa RanBPM. The sequence of the SPRY domain was encountered from the 99 amino acids in AtRanBPM sequence. E- Levels of identities and similarities in amino acid composition of conserved domains between AtRanBPM and its human homologues RanBPM and RanBP10. Click here for file

Additional file 3 **Phylogenetic analysis of AtRanBPM and its homologues from other eukaryotic species.** The tree was constructed by the neighbor-joining method with the MEGA 5.05 software [23]. Branch numbers represent the percentage of bootstrap values in 1000 sampling replicates. The protein accession numbers are [Swiss-prot:F4HYD7] for AtRanBPM At1g35470, [Swiss-prot: Q9SMS1] At4g09340 (segmental genome duplication of chromosome 1), [Swiss-Prot:B9S762] for *R. communis*, [Swiss-Prot:F6HWC3] for *V. vinifera*, [Swiss-Prot:B9MWC1] for *P. trichocarpa*, [Swiss-Prot:C5XUT1] for *S. bicolor*, [Swiss-Prot:Q6ZI83] for *O. sativa*, [Swiss-Prot:B6UAR9] for *Z. mays*, [Swiss-prot: Q6VN20] for human RanBP10, [Swiss-prot: A3KMV8] for RanBP10 from *Bos taurus*, [Swiss-prot: B5LX41] for RanBP10 from *Felis catus*, [Swiss-prot: Q6VN19] for RanBP10 from *Mus musculus*, [Swiss-prot: Q1LUS8] for RanBP10 from *Danio rerio*, [Swiss-prot: Q9PTY5] for RanBP9 from *Xenopus laevis*, [Swiss-prot: Q96S59] for human RanBP9, [Swiss-prot: P69566] for RanBP9 from *Mus musculus*, [Swiss-prot: Q4Z8K6] for RanBP9/10 from *Drosophila melanogaster* and [Swiss-prot: P53076] for Gid1/Vid30 homologue from *Saccharomyces cerevisiae*. Distance bars are given bottom left and bootstrap values are indicated at the nodes. Click here for file

Additional file 4 **Immunopurification of GFP-AtRanBPM protein.** Immunopurification of GFP-AtRanBPM from extracts of GFP-AtRanBPM expressing cell cultures (IP GFP-RanBPM). GFP immunopurification from extracts of wild type Ler *Arabidopsis* cells (IP WT) was used as a negative control. A- Proteins were silver stained after separation on SDS-PAGE. Bands corresponding to MW similar of the proteins copurified with GFP-AtRanBPM (IP GFP-RanBPM) were not present in the negative control (IP WT). B- Signal for AtRanBPM was absent in the negative control (IP WT) after detection with anti-AtRanBPM antibody on Western blots. C- Proteins identified by MALDI-MS in negative control (IP WT in A) were background contamination. Click here for file

Additional file 5 **Identities and similarities between proteins copurifying with AtRanBPM and human CTLH complex members.** Identities and similarities between *Arabidopsis* and human proteins were analysed in WU-BLAST. Click here for file

Additional file 6 **Additional proteins copurified with AtRanBPM.** The proteins were identified by LC-MALDI-MS/MS and the identity of the matched peptides was confirmed by high-resolution MALDI-FTMS with mass accuracy below 1 ppm. Click here for file

Additional file 7 **GFP-AtRanBPM in*Arabidopsis*root cells.** AtRanBPM-GFP signal is dynamic and moving with the cytoplasmic stream. Click here for file

Additional file 8 **Cellular localization of C-terminal GFP and N-terminal GFP AtRanBPM fusion proteins.** Cells of *Arabidopsis* expressing C-terminal GFP AtRanBPM (AtRanBPM-GFP) showed weak cytoplasmic and nuclear signal and accumulation of perinuclear GFP signal similarly as observed for N-terminal GFP AtRanBPM (GFP-AtRanBPM). Click here for file
