# Supplementary material for: Interactions of an Arabidopsis RanBPM homologue with LisH-CTLH domain proteins revealed high conservation of CTLH complexes in eukaryotes
Source: BMC Plant Biol. 2012 Jun 7;12:83. doi: 10.1186/1471-2229-12-83 (PMC3464593; doi:10.1186/1471-2229-12-83)
Supplement: Additional file 1 — Multiple sequence alignment of AtRanBPM plant homologues. Sequence alignment of Arabidopsis RanBPM with Ricinus communisVitis viniferaPopulus trichocarpaSorghum bicolorOryza sativa and Zea mays homologues. Alignment was done using ClustalX2 software [59]. Sequence data of this alignment can be found at accession numbers [Swiss-Prot:F4HYD7] for At1g35470, [Swiss-Prot:B9S762] for R. communis, [Swiss-Prot:F6HWC3] for V. vinifera, [Swiss-Prot:B9MWC1] for P. trichocarpa, [Swiss-Prot:C5XUT1] for S. bicolor, [Swiss-Prot:Q6ZI83] for O. sativa, [Swiss-Prot:B6UAR9] for Z. mays. [file 1471-2229-12-83-S1.pdf]

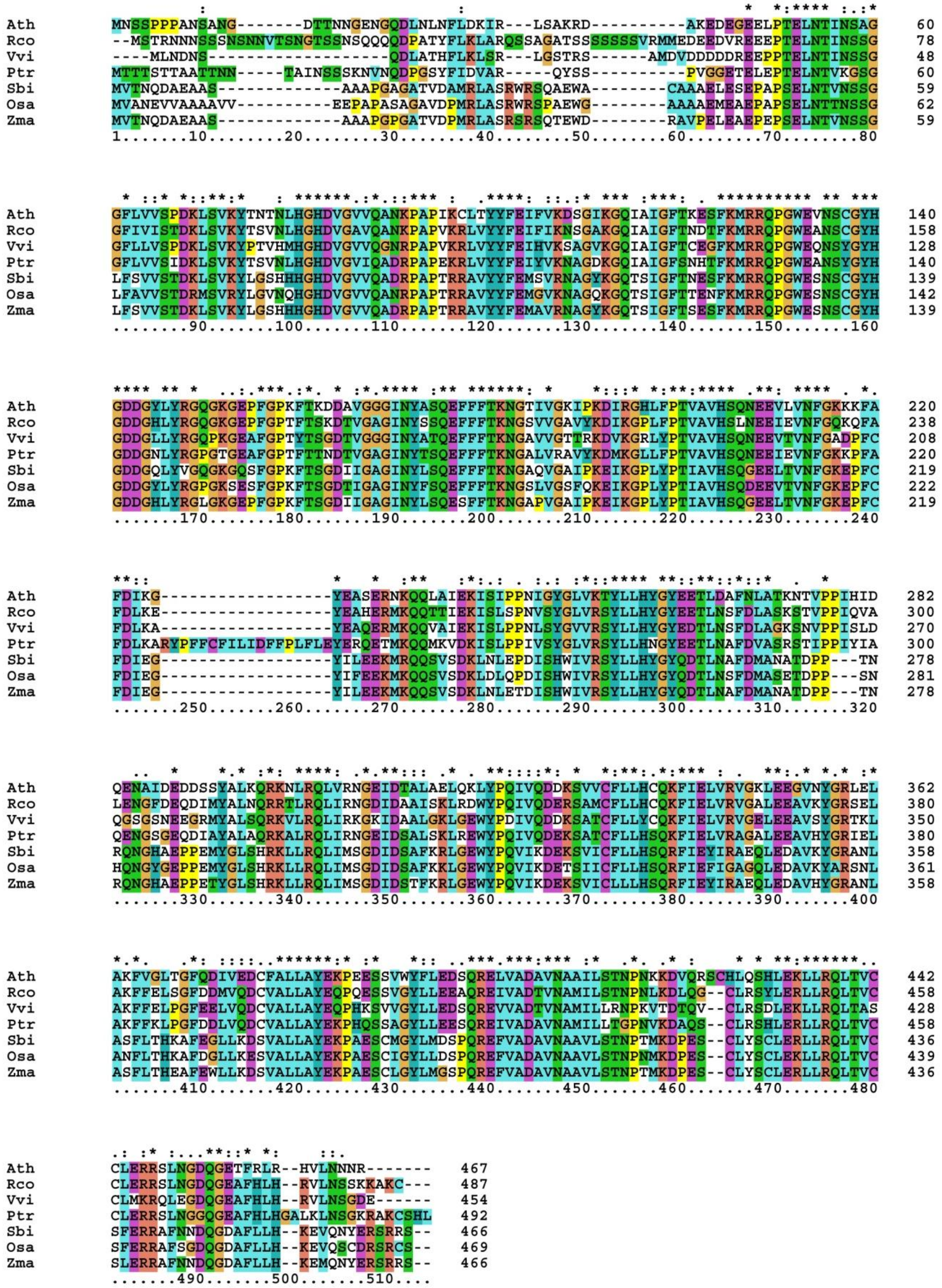

Additional file 1: **Multiple sequence alignment of AtRanBPM plant homologues**. Sequence alignment of *Arabidopsis* RanBPM with *Ricinus communis*, *Vitis vinifera*, *Populus trichocarpa*, *Sorghum bicolor*, *Oryza sativa* and *Zea mays* homologues. Alignment was done using ClustalX2 software (Larkin et al., 2007). Sequence data of this alignment can be found at accession numbers [Swiss-Prot:F4HYD7] for At1g35470, [Swiss-Prot:B9S762] for *R.*

communis, [Swiss-Prot:F6HWC3] for *V. vinifera*, [Swiss-Prot:B9MWC1] for *P. trichocarpa*, [Swiss-Prot:C5XUT1] for *S. bicolor*, [Swiss-Prot:Q6ZI83] for *O. sativa*, [Swiss-prot:B6UAR9] for *Z. mays*.
