# Supplementary material for: Interactions of an Arabidopsis RanBPM homologue with LisH-CTLH domain proteins revealed high conservation of CTLH complexes in eukaryotes
Source: BMC Plant Biol. 2012 Jun 7;12:83. doi: 10.1186/1471-2229-12-83 (PMC3464593; doi:10.1186/1471-2229-12-83)
Supplement: Additional file 2 — Identities and similarities of conserved domains between Arabidopsis RanBPM and its human homologues. Sequence alignment was done for conserved domains SPRY (A), LisH (B), CTLH (C) and CRA (D) of full-sized 90 kDa form (RanBPM_90) and 55 kDa form (RanBPM_55) of human RanBPM protein, and RanBP10 with Arabidopsis RanBPM using ClustalX2 software [59]. Sequence data of this alignment can be found at accession numbers [Swiss-Prot:Q6VN20] for RanBP10, [Swiss-Prot:Q96S59] for 90 kDa RanBPM and [EMBL:BAA23216] for 55 kDa RanBPM. The sequence of the SPRY domain was encountered from the 99 amino acids in AtRanBPM sequence. E- Levels of identities and similarities in amino acid composition of conserved domains between AtRanBPM and its human homologues RanBPM and RanBP10. [file 1471-2229-12-83-S2.pdf]

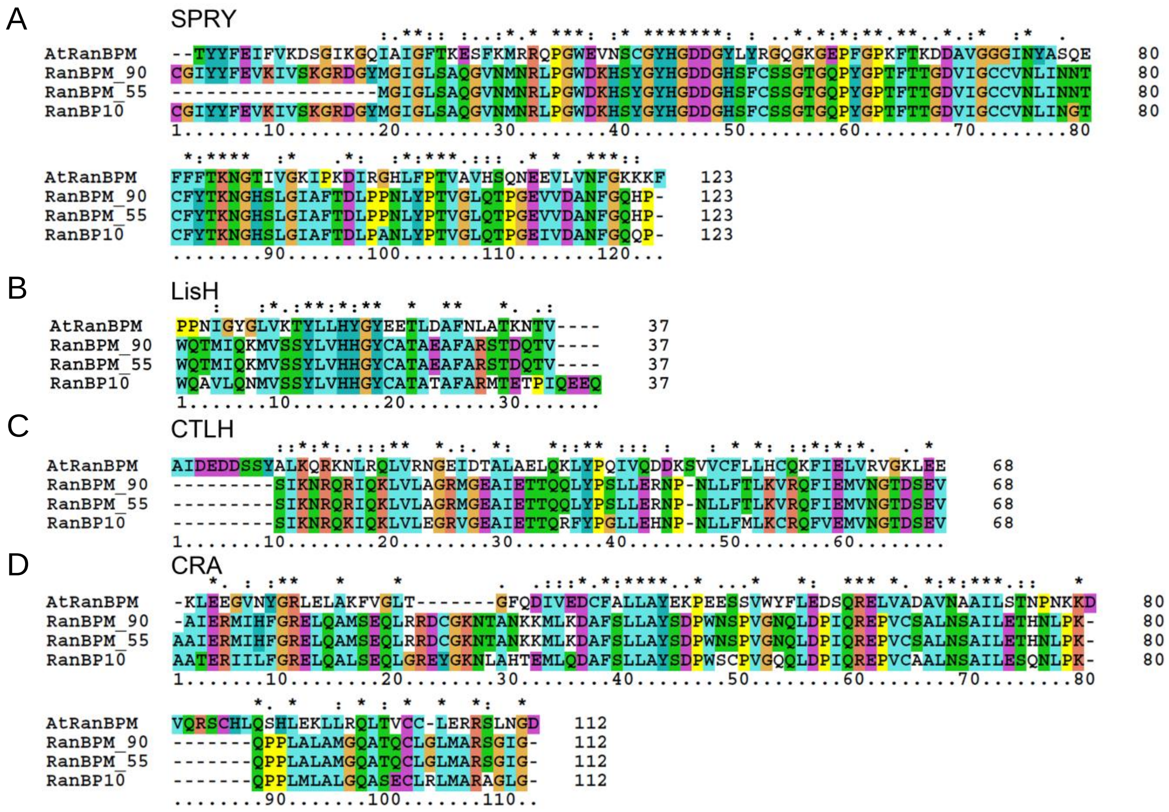

E

	RanBPM		RanBP10	
	Identities [%]	Similarities [%]	Identities [%]	Similarities [%]
**SPRY**	40,0	58,3	39,7	58,7
**LisH**	46,2	69,2	43,5	65,2
**CTLH**	30,8	67,3	28,8	59,2
**CRA**	43,8	62,5	41,9	62,8

Additional file 2: **Identities and similarities of conserved domains between *Arabidopsis* RanBPM and its human homologues**. Sequence alignment was done for conserved domains SPRY (A), LisH (B), CTLH (C) and CRA (D) of full-sized 90 kDa form (RanBPM_90) and 55 kDa form (RanBPM_55) of human RanBPM protein, and RanBP10 with *Arabidopsis* RanBPM using ClustalX2 software [58]. Sequence data of this alignment can be found at accession numbers [Swiss-Prot:Q6VN20] for RanBP10, [Swiss-Prot:Q96S59] for 90 kDa RanBPM and [EMBL:BAA23216] for 55 kDa RanBPM. Sequence of SPRY domain was encountered from the 99 amino acid in AtRanBPM sequence. E- Levels of identities and

similarities in amino acid composition of conserved domains between AtRanBPM and its human homologues RanBPM and RanBP10.
