# Supplementary material for: Interactions of an Arabidopsis RanBPM homologue with LisH-CTLH domain proteins revealed high conservation of CTLH complexes in eukaryotes
Source: BMC Plant Biol. 2012 Jun 7;12:83. doi: 10.1186/1471-2229-12-83 (PMC3464593; doi:10.1186/1471-2229-12-83)
Supplement: Additional file 3 — Phylogenetic analysis of AtRanBPM and its homologues from other eukaryotic species. The tree was constructed by the neighbor-joining method with the MEGA 5.05 software [23]. Branch numbers represent the percentage of bootstrap values in 1000 sampling replicates. The protein accession numbers are [Swiss-prot:F4HYD7] for AtRanBPM At1g35470, [Swiss-prot: Q9SMS1] At4g09340 (segmental genome duplication of chromosome 1), [Swiss-Prot:B9S762] for R. communis, [Swiss-Prot:F6HWC3] for V. vinifera, [Swiss-Prot:B9MWC1] for P. trichocarpa, [Swiss-Prot:C5XUT1] for S. bicolor, [Swiss-Prot:Q6ZI83] for O. sativa, [Swiss-Prot:B6UAR9] for Z. mays, [Swiss-prot: Q6VN20] for human RanBP10, [Swiss-prot: A3KMV8] for RanBP10 from Bos taurus, [Swiss-prot: B5LX41] for RanBP10 from Felis catus, [Swiss-prot: Q6VN19] for RanBP10 from Mus musculus, [Swiss-prot: Q1LUS8] for RanBP10 from Danio rerio, [Swiss-prot: Q9PTY5] for RanBP9 from Xenopus laevis, [Swiss-prot: Q96S59] for human RanBP9, [Swiss-prot: P69566] for RanBP9 from Mus musculus, [Swiss-prot: Q4Z8K6] for RanBP9/10 from Drosophila melanogaster and [Swiss-prot: P53076] for Gid1/Vid30 homologue from Saccharomyces cerevisiae. Distance bars are given bottom left and bootstrap values are indicated at the nodes. [file 1471-2229-12-83-S3.pdf]

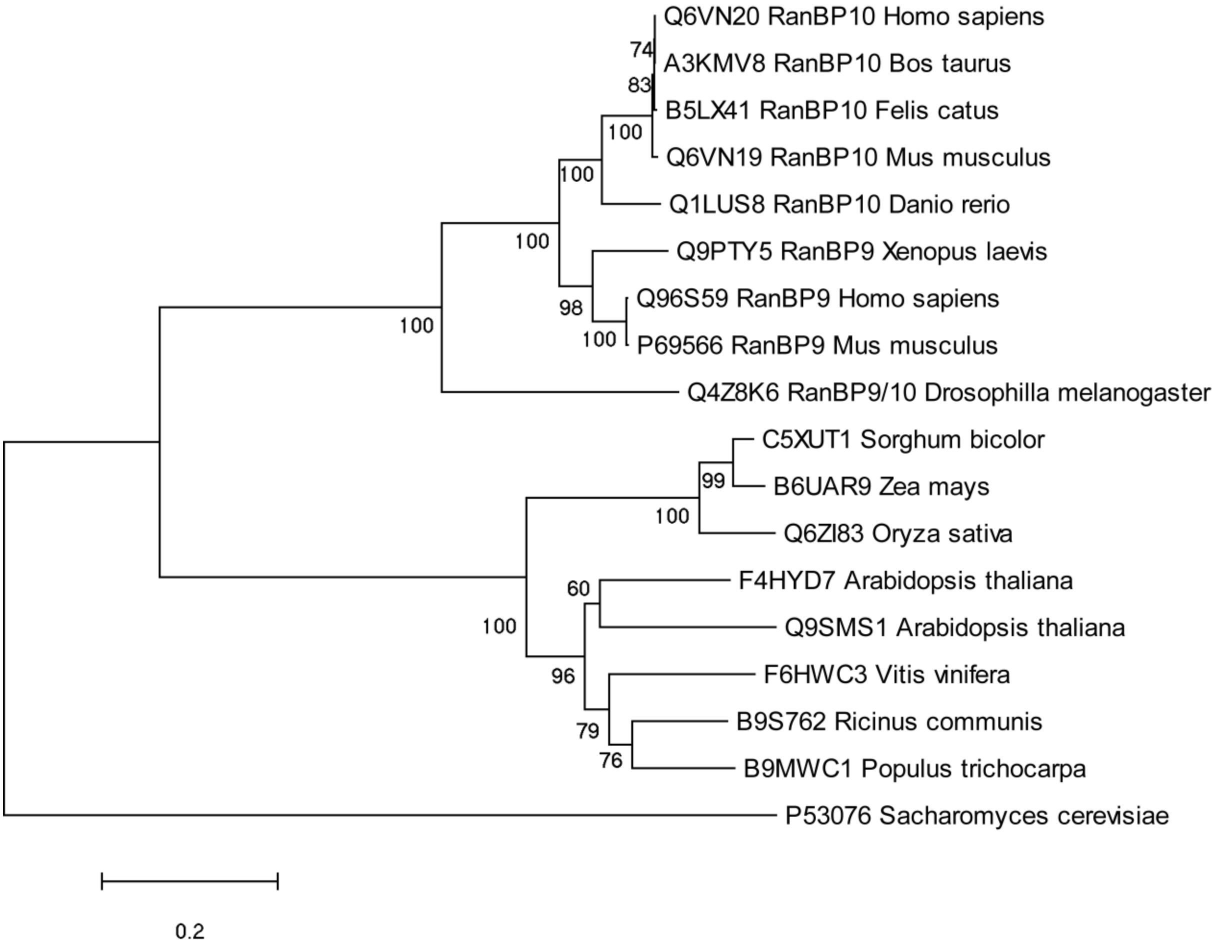

Additional file 3: Phylogenetic analysis of AtRanBPM and its homologues from other eukaryotic species. The tree was constructed by the neighbor-joining method with the MEGA 5.05 program. Branch numbers represent the percentage of bootstrap values in 1000 sampling replicates. The protein accession numbers are [Swiss-prot:F4HYD7] for AtRanBPM At1g35470, [Swiss-prot: Q9SMS1] At4g09340 (segmental genome duplication of chromosome 1), [Swiss-Prot:B9S762] for *R. communis*, [Swiss-Prot:F6HWC3] for *V. vinifera*, [Swiss-Prot:B9MWC1] for *P. trichocarpa*, [Swiss-Prot:C5XUT1] for *S. bicolor*, [Swiss-Prot:Q6ZI83] for *O. sativa*, [Swiss-Prot:B6UAR9] for *Z. mays,* [Swiss-prot: Q6VN20] for human RanBP10, [Swiss-prot: A3KMV8] for RanBP10 from *Bos taurus,* [Swiss-prot: B5LX41] for RanBP10 from *Felis catus,* [Swiss-prot: Q6VN19] for RanBP10 from *Mus musculus,* [Swiss-prot: Q1LUS8] for RanBP10 from *Danio rerio,* [Swiss-prot: Q9PTY5] for RanBP9 from *Xenopus laevis*, [Swiss-prot: Q96S59] for human RanBP9, [Swiss-prot: P69566] for RanBP9 from *Mus musculus*, [Swiss-prot: Q4Z8K6] for RanBP9/10 from *Drosophilla melanogaster* and [Swiss-prot: P53076] for Gid1/Vid30 homologue from *Sacharomyces cerevisiae*. Distance bars are given bottom left and bootstrap values are indicated at the nodes.
