# Supplementary material for: Interactions of an Arabidopsis RanBPM homologue with LisH-CTLH domain proteins revealed high conservation of CTLH complexes in eukaryotes
Source: BMC Plant Biol. 2012 Jun 7;12:83. doi: 10.1186/1471-2229-12-83 (PMC3464593; doi:10.1186/1471-2229-12-83)
Supplement: Additional file 4 — Immunopurification of GFP-AtRanBPM protein. Immunopurification of GFP-AtRanBPM from extracts of GFP-AtRanBPM expressing cell cultures (IP GFP-RanBPM). GFP immunopurification from extracts of wild type Ler Arabidopsis cells (IP WT) was used as a negative control. A- Proteins were silver stained after separation on SDS-PAGE. Bands corresponding to MW similar of the proteins copurified with GFP-AtRanBPM (IP GFP-RanBPM) were not present in the negative control (IP WT). B- Signal for AtRanBPM was absent in the negative control (IP WT) after detection with anti-AtRanBPM antibody on Western blots. C- Proteins identified by MALDI-MS in negative control (IP WT in A) were background contamination. [file 1471-2229-12-83-S4.pdf]

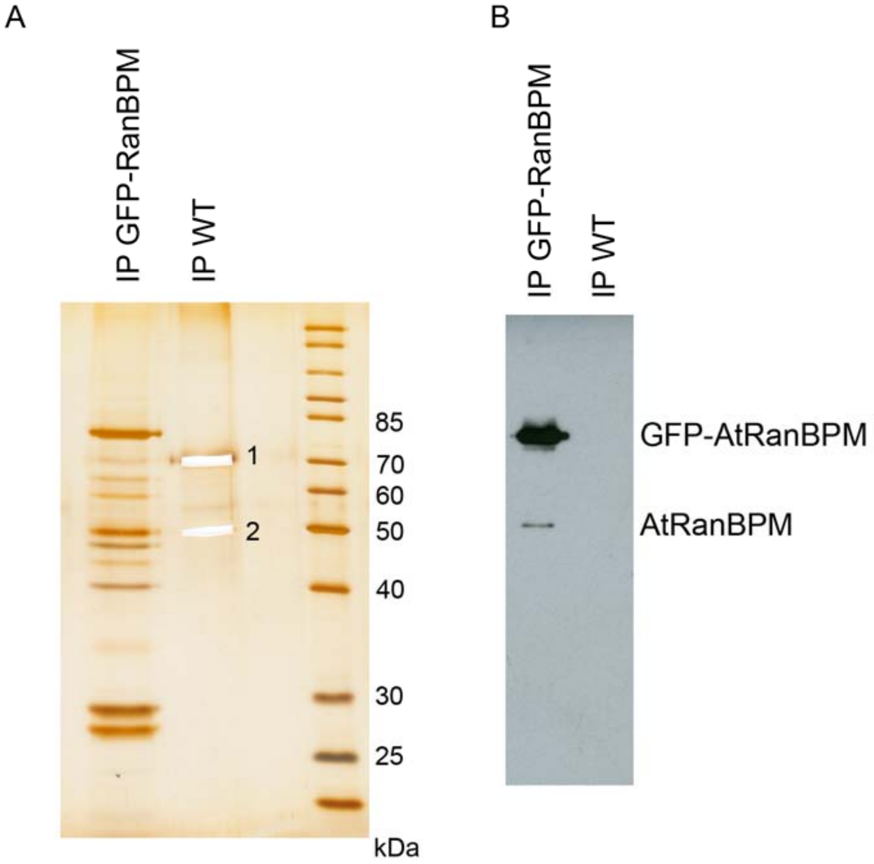

C

	Protein name	DTB No.	MW [kDa]	No. peptides	Sequence coverage [%]	MSMS confirmation
1	Protein S100-A8	S10A8_HUMAN	11	2	11	LLETECPQYIR
1	Actin, cytoplasmic 1	ACTB_HUMAN	42	4	17	SYELPDGQVITIGNER
1	Keratin, type II cytoskeletal 1	K2C1_HUMAN	66	4	12	No
1	Keratin, type I cytoskeletal 14	K1C14_HUMAN	51	1	1	IRDWYQR

Additional file 4: Immunopurification of GFP-AtRanBPM protein.
Immunopurification of GFP-AtRanBPM from extracts of GFP-AtRanBPM expressing cell cultures (IP GFP-RanBPM). GFP immunopurification from extracts of wild type Ler *Arabidopsis* cells (IP WT) was used as a negative control. A- Proteins were silver stained after separation on SDS-PAGE. Bands corresponding to MW similar of the proteins copurified with GFP-AtRanBPM (IP GFP-RanBPM) were not present in the negative control (IP WT). B- Signal for AtRanBPM was absent in the negative control (IP WT) after detection with anti-AtRanBPM antibody on Western blots. C- Proteins identified by MALDI-MS in negative control (IP WT in A) were background contamination.
