# Supplementary material for: Interactions of an Arabidopsis RanBPM homologue with LisH-CTLH domain proteins revealed high conservation of CTLH complexes in eukaryotes
Source: BMC Plant Biol. 2012 Jun 7;12:83. doi: 10.1186/1471-2229-12-83 (PMC3464593; doi:10.1186/1471-2229-12-83)
Supplement: Additional file 5 — Identities and similarities between proteins copurifying with AtRanBPM and human CTLH complex members. Identities and similarities between Arabidopsis and human proteins were analysed in WU-BLAST. [file 1471-2229-12-83-S5.pdf]

Arabidopsis - human	**At1g35470 - RanBPM**	**At3g55070 - MAEA**	**At4g37880 - RMD5**	**At1g61150 - Twa1**	**At5g08560 - WDR26**	**At5g43920 - WDR26**
**Identities [%]**	31	36	37	42	39	40
**Similarities [%]**	50	58	59	63	59	58

Additional file 5: Identities and similarities between proteins copurifying with AtRanBPM and human CTLH complex members. Identities and similarities between *Arabidopsis* and human proteins were analyzed in WU-BLAST.
