# Supplementary material for: Interactions of an Arabidopsis RanBPM homologue with LisH-CTLH domain proteins revealed high conservation of CTLH complexes in eukaryotes
Source: BMC Plant Biol. 2012 Jun 7;12:83. doi: 10.1186/1471-2229-12-83 (PMC3464593; doi:10.1186/1471-2229-12-83)
Supplement: Additional file 6 — Additional proteins copurified with AtRanBPM. The proteins were identified by LC-MALDI-MS/MS and the identity of the matched peptides was confirmed by high-resolution MALDI-FTMS with mass accuracy below 1 ppm. [file 1471-2229-12-83-S6.pdf]

Protein name	AGI number	MW [kDa]	No. peptides	Sequence coverage [%]	Mascot score
**LisH/CRA domains containing protein**	At1g06060	25	1	4	32
**Yippee family protein**	At5g53940	15	1	9	71
**Yippee-like protein**	At3g08890	12	1	17	56
**Yippee family protein**	At2g40110	15	1	9	47
**Armadillo/beta-catenin-like repeat-containing protein**	At3g08947	97	4	5	120

Additional file 6: **Additional proteins copurified with AtRanBPM.** The proteins were identified by LC-MALDI-MS/MS and the identity of the matched peptides was confirmed by high-resolution MALDI-FTMS with mass accuracy below 1 ppm.
