# Supplementary material for: Interactions of an Arabidopsis RanBPM homologue with LisH-CTLH domain proteins revealed high conservation of CTLH complexes in eukaryotes
Source: BMC Plant Biol. 2012 Jun 7;12:83. doi: 10.1186/1471-2229-12-83 (PMC3464593; doi:10.1186/1471-2229-12-83)
Supplement: Additional file 8 — Cellular localization of C-terminal GFP and N-terminal GFP AtRanBPM fusion proteins. Cells of Arabidopsis expressing C-terminal GFP AtRanBPM (AtRanBPM-GFP) showed weak cytoplasmic and nuclear signal and accumulation of perinuclear GFP signal similarly as observed for N-terminal GFP AtRanBPM (GFP-AtRanBPM). [file 1471-2229-12-83-S8.pdf]

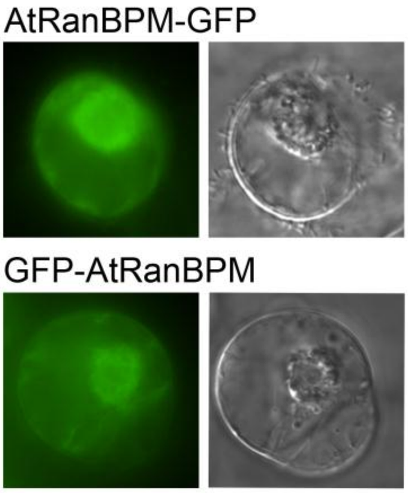

Additional file 8: Cellular localization of C-terminal GFP and N-terminal GFP AtRanBPM fusion proteins.

Cells of *Arabidopsis* expressing C-terminal GFP AtRanBPM (AtRanBPM-GFP) showed weak cytoplasmic and nuclear signal and accumulation of perinuclear GFP signal similarly as observed for N-terminal GFP AtRanBPM (GFP-AtRanBPM).
